# Preliminary Study on Changes of Sleep EEG Power and Plasma Melatonin in Male Patients With Major Depressive Disorder After 8 Weeks Treatment

**DOI:** 10.3389/fpsyt.2021.736318

**Published:** 2021-11-12

**Authors:** Xue-Qin Wang, De-Quan Wang, Yan-Ping Bao, Jia-Jia Liu, Jie Chen, Shao-Wei Wu, Hsuan-Nu Luk, Ling Yu, Wei Sun, Yong Yang, Xue-Hua Wang, Lin Lu, Jia-Hui Deng, Su-Xia Li

**Affiliations:** ^1^NHC Key Laboratory of Mental Health (Peking University), National Clinical Research Center for Mental Disorders (Peking University Sixth Hospital), Peking University Institute of Mental Health, Peking University Sixth Hospital, Beijing, China; ^2^National Institute on Drug Dependence and Beijing Key Laboratory of Drug Dependence, Peking University, Beijing, China; ^3^Department of Occupational and Environmental Health Sciences, School of Public Health, Peking University, Beijing, China; ^4^Key Laboratory of Molecular Cardiovascular Sciences, Peking University, Ministry of Education, Beijing, China; ^5^Peking University Health Science Center, Beijing, China; ^6^School of Automation, Hangzhou Dianzi University, Hangzhou, China; ^7^Beijing United Family Hospital, Beijing, China; ^8^Peking-Tsinghua Center for Life Sciences and PKU-IDG/McGovern Institute for Brain Research, Peking University, Beijing, China; ^9^Department of Pharmacology, School of Basic Medical Sciences, Peking University Health Science Center, Beijing, China

**Keywords:** major depressive disorder, polysomnography, escitalopram, melatonin, sleep EEG power

## Abstract

**Objective:** To clarify the effects of escitalopram on sleep EEG power in patients with Major depressive disorder (MDD).

**Method:** Polysomnography (PSG) was detected overnight, and blood samples were collected at 4 h intervals over 24 h from 13 male healthy controls and 13 male MDD patients before and after treatment with escitalopram for 8 weeks. The outcome measures included plasma melatonin levels, sleep architecture, and the sleep EEG power ratio.

**Results:** Compared with healthy controls, MDD patients presented abnormalities in the diurnal rhythm of melatonin secretion, including peak phase delayed 3 h and a decrease in plasma melatonin levels at night and an increase at daytime, accompanied by sleep disturbances, a decrease in low-frequency bands and an increase in high-frequency bands, and the dominant right-side brain activity. Several of these abnormalities (abnormalities in the diurnal rhythm of melatonin secretion, partial sleep architecture parameters) persisted for at least the 8-week testing period.

**Conclusions:** Eight weeks of treatment with escitalopram significantly improved subjective sleep perception and depressive symptoms of patients with MDD, and partially improved objective sleep parameters, while the improvement of circadian rhythm of melatonin was limited.

## Introduction

Major depressive disorder (MDD) is one of the most prevalent mental health conditions and the leading cause of disability worldwide (1). A meta-analysis found that all antidepressants are more effective than placebo in treating acute MDD in adults, but the treatment response is only moderately effective on average (2). The rates of pharmacotherapy-associated relapse to depression are ~50-80% (3). In patients with remitted depression, 66.6% have residual sleep disturbances (4) that are linked to poorer outcomes (5) and a higher risk of suicide (6, 7). Therefore, the assessment and treatment of sleep disorders in patients with MDD should be paid more attention during clinical treatment.

Previous study demonstrated that one of the core features of mood disorders is disruptions in sleep and circadian rhythmicity, meanwhile individuals with this core feature may be associated with a greater susceptibility to mood disorders (8). Sleep disturbances are common in depressed patients (9), including a shorter latency to rapid-eye-movement (REM) sleep, increased REM sleep (10), impairments in sleep maintenance, decreased slow-wave sleep (SWS) (11), which were assessed by visually scored polysomnography (PSG). Computer-analyzed sleep electroencephalography (EEG) abnormalities, particularly deficient delta activity in non-REM (NREM) sleep, known as slow-wave activity (SWA), had also been reported in MDD (12, 13). Although traditional PSG has provided valuable descriptions of sleep, stage-scoring algorithms do not provide the information of EEG frequency characteristics or rhythmicity that underlie sleep disturbances (12). A previous study indicated that spectrum power analyses of EEGs provide a more complete record of brain electrical activity during sleep (14). However, few studies have evaluated changes in EEG power before and after treatment in patients with MDD. Asymmetries of electrical activity in both hemispheres have been reported, especially alpha and delta power asymmetry, in patients with MDD (15, 16). A previous study revealed the relationship between emotion and hemispheric asymmetry from the perspective of brain networks based on resting state EEG (17). Unfortunately, these findings were not based on analyses of sleep EEGs. Few studies have investigated whether each frequency band of sleep EEGs is asymmetrical.

Circadian rhythm disorder is a very obvious feature in patients with MDD (18). It mainly manifests as a sleep-wake rhythm disorder and melatonin secretion rhythm disorder, with abnormal body temperature regulation (19, 20). Melatonin release time is used as the rhythm marker to determine the circadian phase and period (21). Our previous study showed that patients with MDD had disturbances in melatonin release (19). Another study showed phase advances or delays in the rhythm of melatonin secretion in patients with MDD (22). Whether the variation of melatonin secretion rhythm parameters is correlated with the variation of power ratio in each frequency band of sleep EEG has not been reported.

Medications can have a significant effect on sleep, resulting in either positive or negative effects on sleep (23). Selective serotonin reuptake inhibitors (SSRIs) have an inhibitory effect on REM sleep, with a less consistent impact on NREM (24). Escitalopram, a highly selective SSRI, is the *S*-enantiomer of citalopram with therapeutic activity, and has a particularly marked efficacy and tolerability compared with other antidepressants (25, 26). Previous study showed the improvement in subjective sleep quality after escitalopram administration in a variety of settings (27). Thus, we hypothesized that the remarkable effect of escitalopram in the treatment of depression may be related to the improvement of sleep EEG power in patients.

The main aim of this study is to elucidate the effects of escitalopram on sleep EEG power in patients with MDD. We tested the sleep EEG with PSG, melatonin in peripheral blood by enzyme-linked immunosorbent assay (ELISA). We also analyzed correlations between changes in the EEG power ratio and changes in plasma melatonin rhythm and changes in clinical symptoms in MDD patients.

## Methods

### Design and Participants

Patients with MDD were screened at Peking University Sixth Hospital, Beijing, China, between March 2014 and March 2018. Healthy controls (HCs) were recruited through advertisements in the community. Patients with MDD met the following inclusion criteria: (1) *Diagnostic and Statistical Manual of Mental Disorders*, 5th edition (DSM-5), criteria for MDD and without a history of other mental disorders (the diagnosis was established using the Structured Clinical Interview of the Mini International Neuropsychiatric Interview [MINI], which was performed by two experienced psychiatrists), (2) 17-item Hamilton Rating Scale for Depression (HRSD-17) total score ≥ 22, (3) male patients aged 18-45, (4) right-handed, and (5) 18 ≤ Body Mass Index <30. The exclusion criteria included the following: (1) current or past serious physical illness (e.g., active tuberculosis, acute hepatitis, cirrhosis, renal illness, cardiovascular illness, or unstable diabetes), (2) past or present history of psychoactive substance abuse or taking inhibitors of CYP1A2 and CYP2C9 (e.g., fluvoxamine, ciprofloxacin, and propranolol), (3) caffeinated beverage/pill consumption > 300 mg caffeine daily, (4) worked on shift work within the preceding year, (5) jet lag travel during the last 2 weeks, (6) smoking, (7) significant suicide risk (HRSD suicide score > 2) (8) accompanied by psychiatric symptoms, (9) treatment with modified electroconvulsive therapy (MECT) within 6 months, and (10) refractory depression.

All of the HCs were screened for psychiatric disorders according to the MINI and an interview that was performed by a psychiatrist, and provided a full medical history and details on lifestyle and habits. The inclusion criteria for the HC group included the following: (1) self-reported good sleep (e.g., no sleep complaints) and Pittsburgh Sleep Quality Index (PSQI) score <5, (2) HRSD-17 score <7, and (3) 18 ≤ Body Mass Index <30. The exclusion criteria for the HC group were the following: (1) any exclusion criteria for the MDD group, (2) any past or present history of mental illness that met the DSM-5 diagnostic criteria, (3) sleep disturbances, reflected by self-reported total sleep time (TST), (3) sleep disturbances reflected by total PSQI score, (4) sleep latency (SL) > 30 min or apnea hypopnea index (AHI) ≥ 5 according to PSG, (5) shift work, (6) any medication taken in the past 30 days, and (7) any current or past physical diseases will aggravated or reappear if the individual enrolled in the study.

Sixteen male patients with MDD and 15 age- and gender-matched HCs were recruited. Thirteen MDD patients and 13 HCs who completed all of the experimental procedures were included in the final analyses. Participants included in this study were Han Chinese. Each of them signed a written informed consent form before participation. The ethics committee of Peking University Sixth Hospital approved this study.

### Assessments

The 17-item Hamilton Depression Scale (HRSD-17) score was defined as the primary outcome measure. Secondary measures included the Montgomery-Åsberg Depression Rating Scale (MADRS), Clinical Global Impression-Severity scale (CGI-S), and 14-item Hamilton Anxiety scale (HAMA). These instruments were applied at baseline and after 8 weeks of escitalopram treatment in MDD patients. For HCs, these measures were assessed only once. Additionally, the PSQI and Insomnia Severity Index (ISI) was also applied before PSG recordings.

### Treatment

Patients were prescribed escitalopram (manufactured by H. Lundbeck A/S Copenhagen, repackaged by Xian-Janssen Pharmaceutical Ltd) for 8 weeks. For the first week, patients taken 10 mg escitalopram daily (taken after breakfast). From the day 8, patients taken 15 mg escitalopram daily. Some patients need 20 mg daily from the day 15 if the reduction in HRSD score was <20% at the end of 2 weeks. Then the dosage of escitalopram was maintained for 6 weeks.

### Procedures

All MDD patients underwent two experimental sessions. The first session began before the beginning of escitalopram treatment, and the second session occurred at the 8th weekend of treatment. All healthy volunteers underwent one experimental session.

#### Collection of Peripheral Blood Samples

Participants were asked to arrive at the experimental ward for sleep monitoring at least 1 h before the first blood sampling on the morning of the first day and stay there until the last blood sample was collected on the next day. Upon the participant's arrival, an intravenous catheter was inserted into a forearm vein or vein on the back of the hand. Before the first blood sample was collected, participants were allowed to sit in a recliner to adapt to the experimental environment but was not allowed to sleep. Blood samples were collected at 7 time points over a 24 h period. Blood samples were drawn every 4 h at 8:00 AM, 12:00 PM, 4:00 PM, 8:00 PM, 12:00 AM, 4:00 AM, and 8:00 AM the next day. The experimental environment lights were turned off according to the bedtime of the participants' usual sleep habits at 10:30 PM-11:30 PM, and the participants were awakened at 7:00 AM-7:30 AM. The heparinized isotonic saline was continuously infused into the tubing system to keep patent between blood samplings.

During the 24 h blood samples' collection, subjects were free to sleep, but they had to lie in bed from 10:30 PM-11:30 PM on the first day to 7:00 AM-7:30 AM on the second day. The participants took standardized meals and were allowed to watch television, read books, talk to each other, and walk around the ward from 7:00 AM-7:30 AM to 10:30 PM-11:30 PM on the first day. The experimental environment provided all participants environmental lighting under a natural state like when they were at their home. The lights were turned off at 10:30 PM-11:30 PM. Researchers used mini flashlights to draw the blood samples during the lights-off period. Before the last blood sampling of the each experimental session (i.e., baseline and final sessions), the HRSD, MADRS, HAMA, CGI-S, PSQI, and ISI were applied individually to assess depressive, anxiety, and sleep-wake symptoms.

### Sleep Monitoring

Polysomnograms were recorded overnight in sound-attenuated and light- and temperature-controlled rooms. Participants familiarized themselves with the environment at least 15.5 h before sleep monitoring. PSG was recorded between 10:30 PM-11:30 PM and 7:00 AM-7:30 AM for an entire night (8 h). Daytime sleep was forbidden. The PSG was recorded in accordance with the American Academy of Sleep Medicine guidelines (28). The PSG system (Grael Sleep Recording System, Compumedics, Melbourne, Australia) included six EEG leads (F3, F4, C3, C4, O1, and O2, connecting mastoid), bilateral electrooculogram leads, anterior tibialis and submentalis electromyogram leads, and electrocardiogram. Respiratory rhythm monitoring included oronasal airflow, chest and abdominal breathing efficiency, and oxygen saturation. We collected data for TST, sleep latency (SL), the percentage of each sleep stage (N1%, N2%, N3%, and REM%), REM latency (RL), wake after sleep onset (WASO), sleep continuity (SC) and sleep efficiency (SE). Firstly, online band-pass filter processing (0.5–30 Hz) was conducted for sleep monitoring, then the raw EEG data without filtering was exported to EEGLAB for further analyses.

#### Sleep Stage Identification

The sampling rate was 256 Hz, and the filter settings were the following: coded in the MATLAB environment, because the frequency of the power spectral signal of electrical equipment is usually 50, 100 Hz, etc. (29), the notch filter of EEG signal toolbox in EEGLAB is first used to remove the interference of the 50 Hz power frequency signal. Then use a band-pass filter to filter the data at 0.5–30 Hz. Sleep staging EEG was visually scored by a specialized technician who was a registered PSG technologist who was blinded to subject group using revised American Academy of Sleep Medicine 2.2 sleep scoring criteria (30). We rejected data segment when it has large artifact within its 30-s time window. Interpretation of major body movement is based on AASM 2.2. So, the artifact rejection criteria is: major body movement and muscle artifact abscuring the EEG for more than half an epoch to the extent that the sleep stage cannot be determined. This criterion also applies to EEG power analysis.

#### Power Spectral Analysis

EEG data were imported into EEGLAB and epoched into 30 s bins. After artifact rejection, spectral power was calculated by using the fast Fourier transform (FFT) with Hanning window tapering (Compumedics Sleep Study System) on artifact-free segments. We monitored the C3, C4, F3, F4, O1, and O2 electrodes, which cover the frontal, parietal, and occipital regions and are not contaminated by eye movement artifacts. The frequency bands were defined as the following: delta1 (0.5-2 Hz), delta2 (2-4 Hz), theta (4-8 Hz), alpha (8-13 Hz), beta1 (13-18 Hz), beta2 (18-30 Hz), gamma1 (30-50 Hz), and gamma2 (50-100 Hz). Left and right hemisphere electrodes were separately observed, and the data were analyzed across the C3, F3, and O1 and C4, F4, and O2 electrodes.

Data de-noising was performed using the EEGlab toolkit (31). The working frequency (50 Hz) was first removed, and then the band-pass filter was used, with a filtering range of 0.5-100 Hz. The spectral power density was calculated for each 30-s epoch using the FFT method in MATLAB 2016. The data were normalized to minimize individual differences, dividing every frequency power by the total power, thus generating the power ratio.

#### Serum Melatonin Test

Plasma melatonin concentrations were determined using an enzyme-linked immunosorbent assay (IBL International GmbH, Hamburg, Germany). The sensitivity of the assay was ~1.6 pg/ml for melatonin.

### Statistical Analysis

The data analyses were performed using SPSS 20.0 software (SPSS, Chicago, IL, USA). The participants' demographics were characterized using descriptive statistics (means and standard deviations [SDs] or frequencies). Considering possible variance, determined by homogeneity tests, between the HC and MDD groups, continuous data were analyzed using the Mann-Whitney test. The Wilcoxon rank-sum test was used to compare differences between baseline and after 8 weeks of escitalopram treatment. Correlations among different data were analyzed using Pearson's correlation coefficient. Repeated-measures analysis of variance (ANOVA) was used to compare differences in plasma melatonin levels between groups and between baseline and after 8 weeks of treatment. For MDD patients, the ANOVAs had two factors of repeated measures: Day (baseline and final) and Time of Day. For HCs, the ANOVAs had only one factor of repeated measure: Time of Day. Differences between groups were analyzed using two separate ANOVAs that compared HCs with MDD patients on each of the MDD patients' two test days. Significant differences between each time point, determined by ANOVAs, were followed by Tukey's *post-hoc* test. The single-cosinor method was used to assess the circadian rhythms of melatonin. Significant changes in rhythms were determined by rejecting the zero amplitude hypotheses with 95% certainty, reflected by *p*-values that resulted from comparisons of residuals before and after the cosine curve fit. We calculated the mesor (i.e., middle value of the fitted cosine, representing a rhythm-adjusted mean), amplitude (i.e., half of the difference between the minimum and maximum of the fitted cosine function), and standard error (SE) of their dispersions. The seven time-normalized means were also analyzed to detect effects of time using one-way ANOVA for melatonin. The Benjamini-Hochberg procedure was implemented to correct for multiple comparisons. Values of *p* < 0.05 were considered statistically significant.

## Results

### Demographics and Clinical Assessments

**Figure 1:**
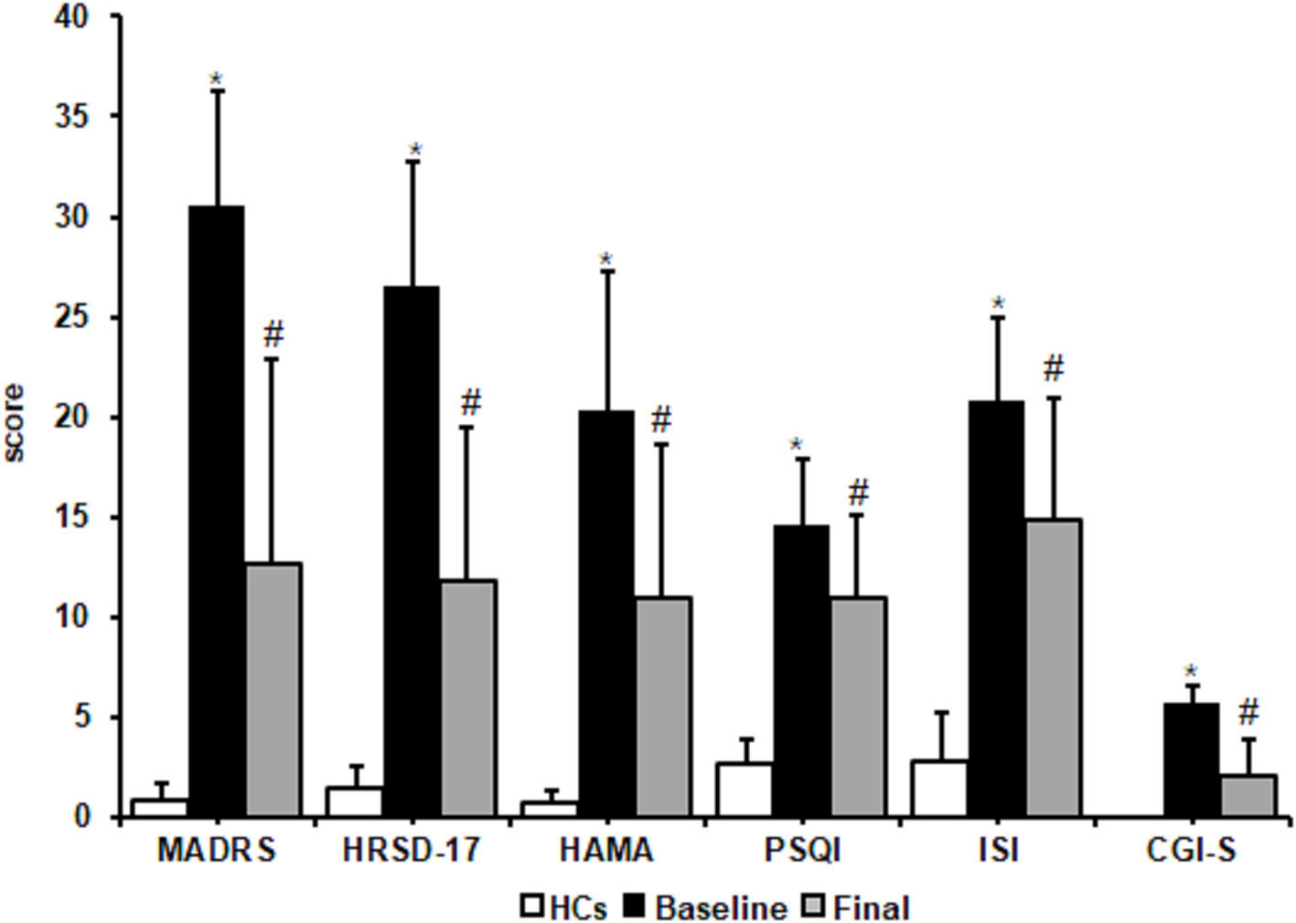
Effects of escitalopram on clinical symptoms. Clinical symptoms significantly decreased after 8 weeks of treatment. MADRS, Montgomery-Asberg Depression Rating Scale; HRSD-17, 17-item Hamilton Rating Scale for Depression; HAMA, Hamilton Anxiety Scale; PSQI, Pittsburgh Sleep Quality Index; ISI, Insomnia Severity Index; CGI-S, Clinical Global impressions-severity. The data are expressed as mean ± SD. *n* = 13 MDD patients. *n* = 13 healthy controls. *p < 0.05, different from healthy controls; ^#^*p* < 0.05, different from baseline. HCs, healthy controls; Baseline, MDD patients before treatment; Final, patients at the end of 8 weeks of escitalopram treatment.

There were no significant differences in age, education, and Body Mass Index between HCs and MDD patients at baseline (Table 1). Patients with MDD who completed the study presented improvements in clinical symptoms. MADRS, HRSD, HAMA, and CGI-S scores significantly decreased after 8 weeks of escitalopram treatment (*p* values < 0.05). The Wilcoxon rank-sum test showed that total PSQI and ISI scores significantly decreased after 8 weeks of treatment (*p* values < 0.05). These results indicated that escitalopram can improve subjective sleep quality, the severity of insomnia symptoms, and depressive symptoms in MDD patients (Figure 1).

**Table 1:** Clinical characteristics of HCs and MDD patients at baseline.

**Variable**	**MDD patients (*****n*** **=** **13)**		**Healthy controls (*****n*** **=** **13)**		***U***	***p* ^**a**^**
	**Mean**	***SD***	**Mean**	***SD***		
Age (years)	31.54	7.25	27.00	4.93	−1.495	0.139
Education (years)	12.77	3.52	13.15	2.70	0.313	0.920
Gender, male (*n* [%])	13 (100%)		13 (100%)			
Body Mass Index (kg/m^2^)	22.61	2.93	23.45	2.56	−0.898	0.390

a *Mann-Whitney test*.

### Sleep Architecture

**Figure 2:**
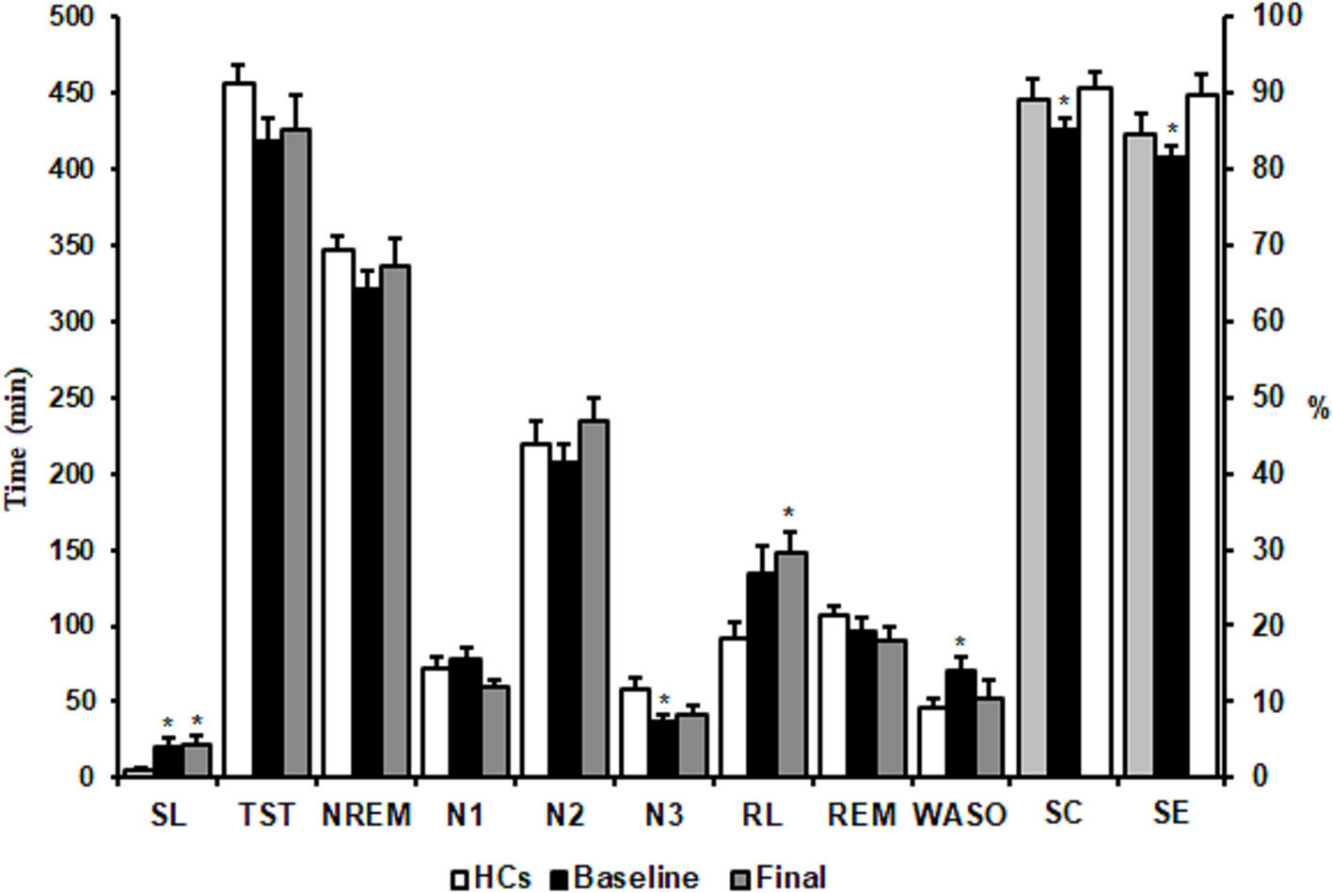
Effects of escitalopram on sleep architectures. SL, sleep latency; TST, total sleep time; NREM, non-rapid-eye-movement; RL, REM sleep latency; REM, rapid-eye-movement; WASO, wake after sleep onset; SE, sleep efficiency; SC, sleep continuity. The data are expressed as mean ± SE. *n* = 13 MDD patients. *n* = 13 healthy controls. *p < 0.05, different from healthy controls. HCs, healthy controls; Baseline, MDD patients before treatment; Final, MDD patients at the end of 8 weeks of escitalopram treatment.

Mann-Whitney comparisons of sleep architectural measures between the HCs and MDD patients are shown in Figure 2. Patients with MDD at baseline had a significant longer SL and WASO, shorter stage 3 NREM sleep, and decreases in SE and SC compared with the HC group (*p* < 0.05). After 8 weeks of escitalopram treatment, SL in patients with MDD was still longer than that in HCs (*p* < 0.05). Unexpectedly, RL in patients with MDD was extended further from at baseline, with a statistically significant difference from the HC group (*p* < 0.05). Other PSG parameters showed trends toward improvement, but these changes were not statistically significant (*p* > 0.05; Figure 2). These results indicated that 8 weeks of treatment with escitalopram only partially improves objective sleep architecture parameters.

### Circadian Melatonin Patterns

Plasma melatonin levels showed significant daily rhythm in HCs [*F*_(6,90)_ = 80.77, *p* = 0.001, cosinor *p* < 0.05] and in patients with MDD at baseline and after 8 weeks of escitalopram treatment [baseline: *F*_(6,90)_ = 86.23, *p* = 0.001, cosinor *p* < 0.05; final: *F*_(6,90)_ = 79.96, *p* = 0.001, cosinor *p* < 0.05]. Peak plasma melatonin levels were observed at 3:00 AM in HCs and at 6:00 AM at baseline and after 8 weeks of escitalopram treatment in patients with MDD. The peak phase was delayed for 3 h in patients with MDD, although escitalopram treatment attenuated symptoms of depression.

**Figure 3:**
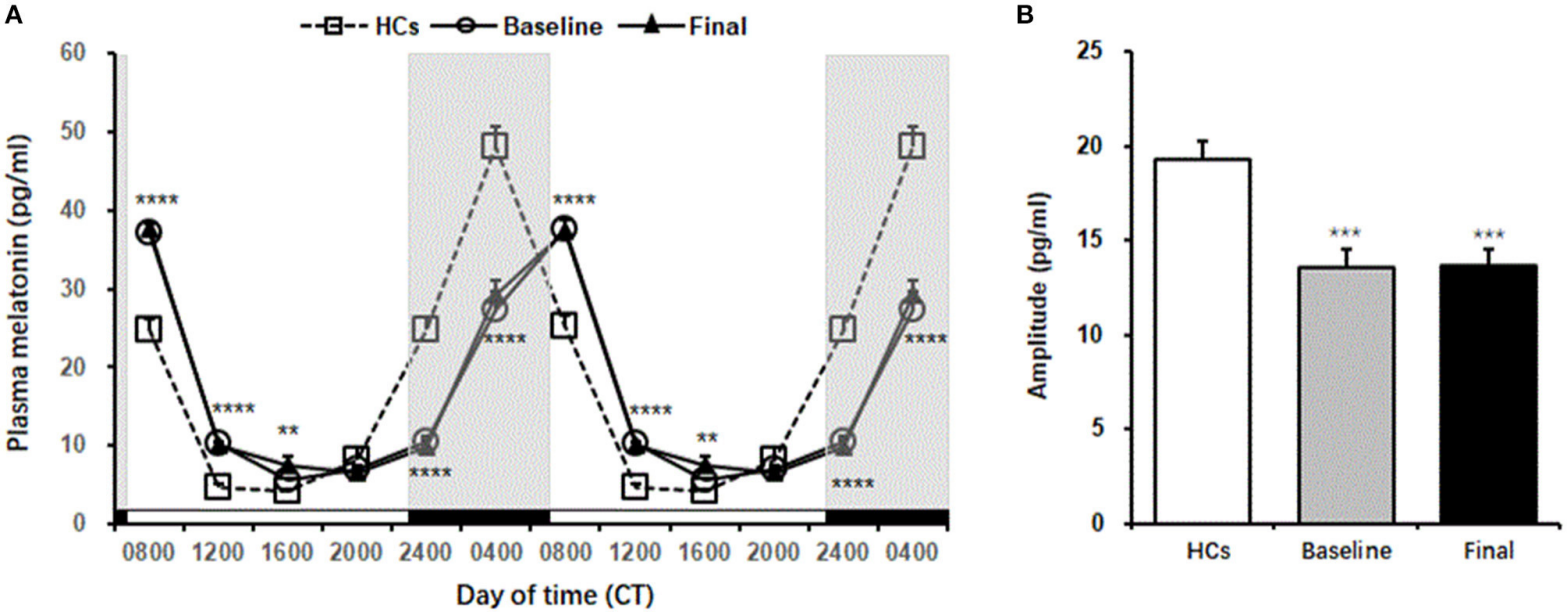
Plasma melatonin levels in healthy controls and MDD patients. **(A)** The melatonin secretion rhythm, which is double-plotted. Shaded regions indicate nighttime. **(B)** Amplitude of melatonin in healthy controls and MDD patients. The data are expressed as mean ± SE. *n* = 13 MDD patients. *n* = 13 healthy controls. **p < 0.01, ***p < 0.001, ****p < 0.0001, different from healthy controls. HCs, healthy controls; Baseline, MDD patients before treatment; Final, MDD patients at the end of 8 weeks of escitalopram treatment.

No significant difference in melatonin levels was found between baseline and after 8 weeks of treatment in patients with MDD [*F*_(1,24)_ = 0.25, *p* = 0.63], without a significant Group × Time of Day interaction [*F*_(6,144)_ = 0.47, *p* = 0.83]. No significant difference in melatonin levels was found between HCs and patients with MDD at baseline [*F*_(1,24)_ = 1.16, *p* = 0.29], with a significant Group × Time of Day interaction [*F*_(6,144)_ = 59.99, *p* < 0.001]. No significant difference in melatonin levels was found between HCs and patients with MDD at the end of 8 weeks of treatment [*F*_(1,24)_ = 0.4, *p* = 0.53], with a significant Group × Time of Day interaction [*F*_(6.144)_ = 59.83, *p* < 0.001]. The Tukey *post-hoc* test revealed that melatonin levels in patients with MDD at both baseline and the end of 8 weeks of treatment were significantly higher than that in HCs at 8:00 AM (*p* < 0.0001), 12:00 PM (*p* < 0.0001), and 4:00 PM (*p* < 0.01), whereas melatonin levels were significantly lower than that in HCs at 12:00 AM (*p* < 0.0001) and 4:00 AM (*p* < 0.0001) (Figure 3A, Table 2).

**Table 2:** Circadian properties of melatonin^a^.

	**Health controls**	**MDD patients (baseline)**	**MDD patients (after 8 weeks of escitalopram treatment)**
Mesor (pg/ml)	19.33 ± 1.01	17.66 ± 0.46	18.05 ± 0.61
Amplitude (pg/ml)	19.27 ± 0.97	13.59 ± 0.91^##^	13.69 ± 0.81^##^
Peak phase (CT)	3:00 AM	6:00 AM	6:00 AM
Cosinor *F*^b^	17.6^*^	9.99^*^	9.56^*^

* *p < 0.05, significant difference in amplitude (F-test); Mesor, middle value of the fitted cosine, representing a rhythm-adjusted mean; Amplitude, half of the difference between the minimum and maximum of the fitted cosine function*.

a *Values are mean ± SE*.

b *Single-cosinor method*.

## *p < 0.05, difference from Health controls*.

Mann-Whitney comparison showed that the amplitude of plasma melatonin in patients with MDD was significantly decreased at baseline compared with that in the controls, and 8 weeks of escitalopram treatment did not improve this reduction (*p* < 0.001; Figure 3B).

### EEG Power Spectrum

#### Differences in EEG Power Ratio Between MDD Patients and HCs

**Figure 4:**
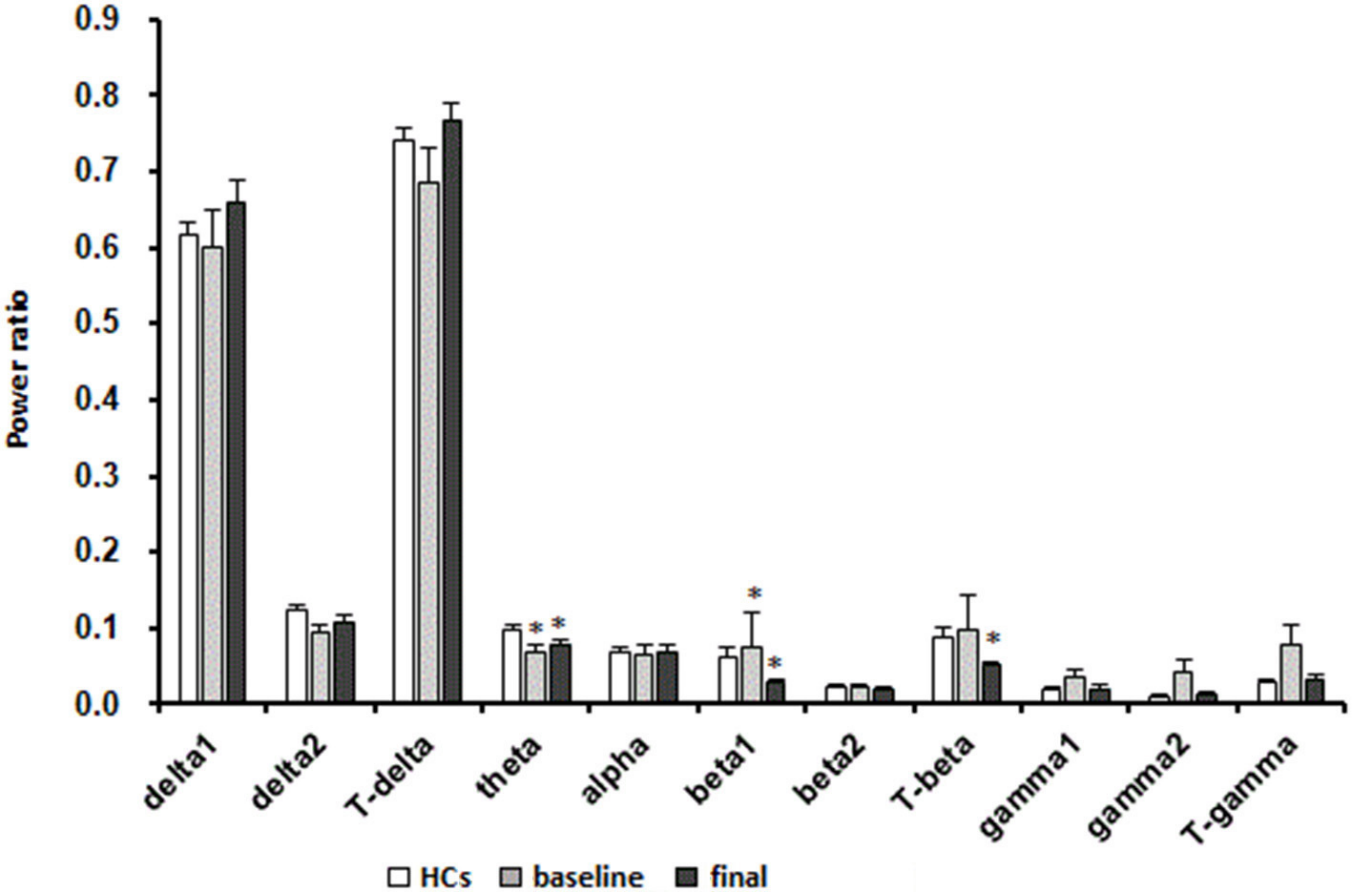
Effects of escitalopram on sleep power measures. The data are expressed as mean ± SE. *n* = 13 MDD patients. *n* = 13 healthy controls. *p < 0.05, different from healthy controls; HCs, healthy controls; Baseline, MDD patients before treatment; Final, MDD patients at the end of 8 weeks of escitalopram treatment.

A significant decrease in the power ratio of the theta frequency band was found in patients with MDD at baseline compared with in HCs (*p* < 0.05, Mann-Whitney test). After 8 weeks of escitalopram treatment, the power ratio of the theta frequency band was still lower than that in HCs (*p* < 0.05, Mann-Whitney test). In patients with MDD, The power ratio of the beta1 frequency band significantly increased at baseline and significantly decreased after 8 weeks of escitalopram treatment compared with that in HCs (*p* < 0.01, Mann-Whitney test). The power ratio of the total beta frequency band significantly decreased in patients with MDD after 8 weeks of escitalopram treatment compared with that in HCs (*p* < 0.01, Mann-Whitney test). Interestingly, the power ratio of the low-frequency band (delta1, delta2, and theta) increased, and the high-frequency band (beta1, beta2, gamma1, and gamma2) decreased after 8 weeks of escitalopram treatment, although these differences were not statistically significant (*p*-values > 0.05; Figure 4).

#### EEG Power Ratio Between Bilateral Cerebral Hemispheres

Supplementary Table 1 shows a significant decrease in the theta frequency band and a significant increase in the total gamma frequency band in the right hemisphere in patients with MDD at baseline compared with that in HCs (*p* < 0.05). After 8 weeks of escitalopram treatment, the theta frequency band in the right hemisphere in patients with MDD was still significantly lower than that in HCs (*p* < 0.05). Significant changes in the EEG power ratios in the left and right hemispheres were seen after 8 weeks of escitalopram treatment. The delta1 frequency band significantly increased, and the delta2 frequency band significantly decreased in the right hemisphere compared with in the left hemisphere in patients with MDD (*p* < 0.05). The theta and alpha frequency bands significantly decreased in the right hemisphere compared with in the left hemisphere in patients with MDD (*p* < 0.05; Supplementary Table 1).

#### Correlations Between Changes in Sleep EEG Power Ratios and Changes in Plasma Melatonin Rhythm and Clinical Symptoms

After 8 weeks of escitalopram treatment in patients with MDD, changes in the power ratio of the delta1 frequency band were negatively correlated with changes in the amplitude of melatonin (*p* < 0.05). The changes in the power ratio of the delta2 and theta frequency bands were positively correlated with mean plasma melatonin levels (*p* < 0.05). The changes in the power ratio of the beta1 and total beta frequency bands were positively correlated with changes in the peak phase of melatonin (*p* < 0.05; Supplementary Table 2). The changes in the power ratios of the gamma1, gamma2, and total gamma frequency bands were positively correlated with changes in HAMA and CGI-S scores in MDD patients (*p* < 0.05; Supplementary Table 3).

## Discussion

The present study showed that 8 weeks of escitalopram treatment significantly improved both depressive symptoms and subjective sleep perception during an acute depression episode. The theta power ratio in the right hemispheres decreased at both baseline and the end of 8 weeks of treatment compared with in HCs. The theta power ratio in the left hemisphere was lower than that in the right hemisphere at baseline. After 8 weeks of escitalopram treatment, the theta power ratio changed to be higher in the left than that in the right. The power ratios of the delta2, theta, and alpha frequency bands switched from being higher in the right hemisphere than in the left hemisphere, although no statistic significance was found between left and right hemisphere at baseline, to being lower in the right hemisphere than in the left hemisphere after 8 weeks of escitalopram treatment. While the power ratio of the delta1 frequency band in the right hemisphere was higher than in the left hemisphere after 8 weeks of escitalopram treatment. Comparation with HCs, the 3-h delay in the peak phase and a significant decrease in the amplitude of melatonin rhythm in MDD patients at baseline did not improved after 8 weeks' treatment with escitalopram. Interestingly, we also found a trend that an increase in the power ratio of low-frequency bands and decrease in the power ratio of high-frequency bands, although the difference was not statistically significant.

### Effects of Escitalopram on Clinical Efficacy and Sleep Architecture

Depressive symptoms, subjective sleep quality and the severity of insomnia were significantly ameliorated after 8 weeks of escitalopram treatment. It's different from subjective assessments, partial improvements in sleep architecture were observed in MDD patients, assessed by PSG. Residual sleep disturbances were still evident at the end of 8 weeks of treatment, when most depressive symptoms improved, including SL and RL in MDD patients were still significantly longer than that in HCs, other parameters of sleep architecture improved, but not statistically significant. This result is consistent with a previous study (32).

Some antidepressants have been reported to improve several sleep variables by increasing sleep efficiency and the amount of REM sleep. Other antidepressants (e.g., tricyclic antidepressants and SSRIs), however, have negative effects on sleep architecture by reducing the duration of REM sleep and increasing RL (33). Additionally, A previous study reported that REM sleep was under circadian control (34). Our results revealed that 8 weeks of escitalopram treatment did not improve the melatonin secretion rhythm, as well as further prolonged RL and decreased the duration of REM sleep in MDD patients, although the latter was not statistically significant. Our results provide partial explanation and further evidences for the limited role of antidepressants in improving objective sleep architecture.

### Effects of Escitalopram on Sleep EEG Power Ratios of Each Frequency Band

Previous studies showed that theta frequency band oscillations are implicated in numerous brain functions, such as voluntary movement, sensory processing, and cognitive function (35, 36). The present findings indicate that the power ratio of the theta frequency band significantly decreased in MDD patients at baseline compared with in HCs. Eight weeks of escitalopram treatment increased the power ratio of the theta frequency band, but this increase was not statistically significant and was still lower than that in HCs. This may explain why many functions, especially cognitive function, are not restored in patients who present the disappearance of clinical symptoms and why the long-term use of antidepressants is needed to prevent recurrence.

Previous study reported that increased beta activity in untreated, actively depressed patients was related to a number of clinical variables, increased beta activity was positively correlated with a recurrent course of depression (37) and negatively correlated with agitation (37) anxiety and symptoms of psychomotor retardation (38, 39) in depression. Additionally, previous study also showed that total-beta (13-30 Hz) activity in cortico-limbic/paralimbic regions decreased after treatment in responders but not in non-responders, suggesting that the normalization of total-beta activity in cortico-limbic/paralimbic regions may be associated with a significant reduction in depressive symptoms (40), which is consistent with our findings that the power ratio of the total-beta frequency band significantly decreased in MDD patients at the end of 8-week escitalopram treatment. Moreover, accumulated researches revealed that beta power increases in parallel with a reduction of cortical excitability (41–43) and with an increase of GABA levels (44–46). These previous studies and our findings can well explain the reason why 8 weeks of escitalopram significantly improved clinical depression symptoms.

Our previous published paper (47) in 2020 that also presented a more thorough analysis of EEG changes in MDD patients before and after escitalopram treatment. Although that paper has a slightly smaller cohort of patients (only 11), and it does not include the data of plasma melatonin level and clinical symptom outcome results. This study is a secondary analysis of the sample in our previous study (47). The analysis of the overnight EEG is across the entire sleep period in the present study and in the previous study we differentiated among various sleep stages (stage1, stage2, stage3, and REM) and the non-linear analysis was also carried out. The analysis of each frequency band is more detailed in the present study. The power ratio of the delta1 frequency band was significantly higher than that before treatment in our previous study (47), while in our present study no significant changes of it was found. Although it seems to be an increase in delta1 after 8 weeks treatment from the Figure 4. This difference may be due to differences in statistical methods. We recognized that using the *t*-test may not be scientific enough for the present study. Although the comparison with the two time points (before and after treatment) can be conducted by paired *t*-test or one-way repeated measure ANOVA, we think the non-parametric tests is more reasonable in the case of uneven variance or non-normal distribution.

### Effects of Escitalopram on Hemispheric Asymmetry

Our findings indicated that the power ratio of the theta frequency band significantly decreased, and the total gamma frequency band significantly increased in the right hemisphere in MDD patients at baseline compared with in HCs. After 8 weeks of escitalopram treatment, the power ratios of the alpha, theta, and delta2 frequency bands in the right hemisphere significantly decreased, and the power ratio of the delta1 frequency band in the right hemisphere significantly increased compared with in the left hemisphere. Previous studies showed that right-sided brain activity, reflected by higher alpha power on the opposite homologous electrode, may be linked to lower sensitivity to reward and lower approach motivation (48–50). Interestingly, previous studies also indicated that the left hemisphere is dominant for positive emotions, and the right hemisphere is dominant for negative emotions (51–55). The present results showed that 8 weeks of escitalopram treatment balanced and reconfigured alpha, theta, and delta2 power between the left and right hemispheres, thus leading to dominance of the left hemisphere. This may explain the amelioration of clinical symptoms of depression, although the power ratio of the other frequency bands was not significantly different between the left and right hemispheres. This condition may also explain why the long-term use of antidepressants can lead to a manic phase. This requires the attention of clinicians and suggests that researchers who work on drug development should seek to avoid such adverse effects of antidepressants.

There have been few studies to examine the hemispheric asymmetry of the power ratio of the delta frequency band, particularly, which is divided into delte1 at lower frequencies and delta2 at higher frequencies. Previous studies showed that right lateralized absolute and relative delta power have been shown to be elevated in depressed individuals compared with controls (56, 57). Our result that the power ratio of the delta2 frequency band in the right hemisphere is lower than that in the left hemisphere after 8–week treatment with escitalopram may explain why escitalopram reduces depressive symptoms. As for the power ratio of the delta1 frequency band in the right hemisphere is higher than that in the left hemisphere after 8–week treatment with escitalopram, no similar results are available. What are the physiological or clinical implications of this finding that are not yet understood. In the future, we will expand the sample size to further study this situation.

Previous studies found that serotonin exerts powerful control over prefrontal gamma rhythm (30-80 Hz) (58), and escitalopram might decrease the inhibitory modulation of serotonin on γ-aminobutyric acid-ergic and glutaminergic neurons, mediated by a reduction of 5-hydroxytryptamine-1A (5-HT_1A_) receptors in limbic regions (59). The power ratio of the total gamma frequency band significantly increased in the right hemisphere in MDD patients at baseline, indirectly suggesting a decrease in serotonin function in the depressed state. The reduction of the power ratio of the total gamma frequency band from baseline to HC levels at the end of 8 weeks of escitalopram treatment might be mediated by long-term postsynaptic 5-HT_1A_ receptor desensitization. We also found that the decrease in the power ratio of the gamma frequency band during sleep was generally positively correlated with the decrease in HAMA total scores and improvements in disease severity which is consistent with previous findings. Several previous studies found that individuals with high levels of anxiety presented increases in gamma-band activity, which might be attributable to the bias of attention toward negative emotional stimuli (60, 61), suggesting gamma-band activity is correlated with the processing of threat perception, attention, and anxiety (62–64).

We also analyzed correlations between changes in melatonin rhythm parameters and changes in sleep EEG power in MDD patients after escitalopram treatment. The changes in the power ratio of the delta2 and theta frequency bands were both positively correlated with changes in the melatonin mesor value, and changes in the power ratio of the delta1 frequency band were negatively correlated with changes in the amplitude of melatonin rhythm oscillation. These results imply that the melatonin secretion rhythm regulates the delta frequency band. Melatonin secretion regulates the delta2 frequency band, and the intensity of the melatonin rhythm regulates the delta1 frequency band. These findings should be replicated in future studies with larger sample sizes, but they may provide a basis for future studies of the ways in which circadian rhythms regulate sleep in humans.

## Limitations

The present study has several limitations. First, only six conventional channels were recorded in the acquisition of EEG during overnight sleep, which limited the possibility of discerning the precise location of changes in the power ratio of each frequency band. Second, the rhythmic expression of peripheral blood leukocyte clock genes does not accurately reflect the rhythmic expression of clock genes in brain tissue. The present study did not detect the rhythmic expression of peripheral blood leukocyte clock genes. Our future animal studies will evaluate the regulation of sleep by circadian rhythm. Third, our sample size was relatively small, and the follow-up time was short. We will increase the sample size and prolong the follow-up time in future studies to clarify the relationship between circadian rhythm and sleep in humans.

## Conclusion

Depressive symptoms, subjective sleep quality, and the severity of insomnia were significantly ameliorated by 8 weeks of escitalopram treatment. The 8 weeks of escitalopram treatment balanced and reconfigured the power ratio of each frequency band in both hemispheres in MDD patients, but the implications of configuration of the delta1 frequency band was not yet understood. Escitalopram has no effect on the delayed peak phase and the decreased amplitude of the melatonin secretion rhythm in MDD patients. In conclusion, the effects of escitalopram were not specific to dysrhythmia. Escitalopram had limited efficacy in improving circadian rhythm and objective parameters of sleep during 8 weeks' treatment. This suggests that further research on the pathogenesis of depression and the development of more targeted antidepressants is still necessary.

## Data Availability Statement

The original contributions presented in the study are included in the article/Supplementary Material, further inquiries can be directed to the corresponding author/s.

## Ethics Statement

The studies involving human participants were reviewed and approved by the Ethics Committee of Peking University Sixth Hospital. The patients/participants provided their written informed consent to participate in this study.

## Author Contributions

X-QW: collect clinical data, formal analysis, and writing—original draft. D-QW, H-NL, and LY: collect PSG data and blood sample. Y-PB: direct data analysis. J-JL and LL: writing—review and editing. JC, S-WW, and X-HW: analyze PSG sleep architecture. WS: patient inclusion and scale evaluation. YY: analyze PSG sleep power. J-HD: analyze PSG sleep architecture and writing—review and editing. S-XL: collect PSG data, blood sample, and writing—review and editing. All authors contributed to the article and approved the submitted version.

## Funding

This work was supported in part by the National Natural Science Foundation of China (nos. 81871071 and 81171251), Capital's Funds for Health Improvement and Research (no. 2014-4-4113), Beijing Municipal Science and Technology Commission (nos. Z161100000516128 and Z181100001718051), and Beijing Municipal Natural Science Foundation (no. 7162101).

## Conflict of Interest

The authors declare that the research was conducted in the absence of any commercial or financial relationships that could be construed as a potential conflict of interest.

## Publisher's Note

All claims expressed in this article are solely those of the authors and do not necessarily represent those of their affiliated organizations, or those of the publisher, the editors and the reviewers. Any product that may be evaluated in this article, or claim that may be made by its manufacturer, is not guaranteed or endorsed by the publisher.
