# Supplementary material for: Preliminary Study on Changes of Sleep EEG Power and Plasma Melatonin in Male Patients With Major Depressive Disorder After 8 Weeks Treatment
Source: Front Psychiatry. 2021 Nov 12;12:736318. doi: 10.3389/fpsyt.2021.736318 (PMC8632954; doi:10.3389/fpsyt.2021.736318)
Supplement: Supplementary file 1 [file Table_1.docx]

Table S1. EEG power ratio between bilateral cerebral hemispheres.

| Variable | Healthy controls  (*n* = 13） | MDD patients (baseline)  (*n* = 13） | MDD patients (after 8 weeks of escitalopram treatment)  (*n* = 13） |
| --- | --- | --- | --- |
| delta1 (Left) | 0.6308 ± 0.0848 | 0.6048 ± 0.1818 | 0.6398 ± 0.1161 |
| delta1 (Right) | 0.6187 ± 0.0707 | 0.5978 ± 0.168 | 0.6781 ± 0.112^#^ |
| delta2 (Left) | 0.1234 ± 0.0198 | 0.0938 ± 0.0442 | 0.113 ± 0.0404 |
| delta2 (Right) | 0.1232 ± 0.0217 | 0.0951 ± 0.0366 | 0.1011 ± 0.0432^#^ |
| Total delta (Left) | 0.7543 ± 0.0796 | 0.6873 ± 0.1843 | 0.7529 ± 0.0494 |
| Total delta (Right) | 0.7419 ± 0.0638 | 0.6819 ± 0.1643 | 0.7792 ± 0.0767 |
| theta (Left) | 0.0923 ± 0.0165 | 0.0671 ± 0.0339 | 0.0827 ± 0.0251 |
| theta (Right) | 0.0971 ± 0.0224 | 0.0703 ± 0.031* | 0.0744 ± 0.0259*^, #^ |
| alpha (Left) | 0.0681 ± 0.0218 | 0.066 ± 0.0498 | 0.0744 ± 0.0553 |
| alpha (Right) | 0.0671 ± 0.0207 | 0.0661 ± 0.0472 | 0.0623 ± 0.0278^#^ |
| beta1 (Left) | 0.0399 ± 0.0349 | 0.0792 ± 0.18 | 0.0319 ± 0.0113 |
| beta1 (Right) | 0.0377 ± 0.0201 | 0.0711 ± 0.1493 | 0.0294 ± 0.0116 |
| beta2 (Left) | 0.0228 ± 0.0154 | 0.0211 ± 0.0122 | 0.0215 ± 0.0142 |
| beta2 (Right) | 0.0233 ± 0.0113 | 0.023 ± 0.0124 | 0.019 ± 0.0107 |
| Total beta (Left) | 0.0627 ± 0.0494 | 0.1003 ± 0.18 | 0.0534 ± 0.0226 |
| Total beta (Right) | 0.0611 ± 0.0305 | 0.0942 ± 0.1486 | 0.0484 ± 0.0204 |
| gamma1 (Left) | 0.0168 ± 0.0064 | 0.0309 ± 0.0384 | 0.021 ± 0.0138 |
| gamma1 (Right) | 0.0214 ± 0.0114 | 0.0408 ± 0.0358 | 0.0201 ± 0.0146 |
| gamma2 (Left) | 0.0076 ± 0.0041 | 0.0353 ± 0.0605 | 0.0128 ± 0.0102 |
| gamma2 (Right) | 0.0132 ± 0.0129 | 0.0504 ± 0.0591 | 0.0136 ± 0.0102 |
| Total gamma (Left) | 0.0243 ± 0.0096 | 0.0662 ± 0.0986 | 0.0339 ± 0.0198 |
| Total gamma (Right) | 0.0346 ± 0.0226 | 0.0912 ± 0.0936* | 0.0336 ± 0.0224 |

Total delta, delta1 + delta2; Total beta, beta1 + beta2; Total gamma, gamma1 + gamma2. The data are expressed as mean ± SD. **p* < 0.05, different from controls (Mann-Whitney test); ^#^*p* < 0.05, different from left hemisphere at the end of 8 weeks of escitalopram treatment (Wilcoxon rank-sum test).

Table S2. Correlation between changes in PSG-EEG power ratio and changes in plasma melatonin rhythm in MDD patients*^a^*.

| Changes in PSG-EEG power ratio | Correlation coefficient | | | | | |
| --- | --- | --- | --- | --- | --- | --- |
|  | Changes in melatonin rhythm (*n* = 13) | | | | | |
|  | Mesor | *p* | Amplitude | *p* | Peak phase | *p* |
| delta1 | -0.314 | 0.297 | -0.632* | 0.020 | -0.270 | 0.372 |
| delta2 | 0.573* | 0.041 | -0.050 | 0.870 | -0.125 | 0.684 |
| Total delta | -0.117 | 0.704 | -0.540 | 0.057 | -0.356 | 0.233 |
| theta | 0.556* | 0.049 | 0.013 | 0.966 | -0.061 | 0.843 |
| alpha | 0.511 | 0.074 | 0.251 | 0.408 | -0.043 | 0.890 |
| beta1 | -0.155 | 0.614 | 0.482 | 0.095 | 0.607* | 0.028 |
| beta2 | 0.121 | 0.693 | -0.305 | 0.311 | -0.214 | 0.482 |
| Total beta | -0.143 | 0.641 | 0.507 | 0.077 | 0.590* | 0.034 |
| gamma1 | 0.132 | 0.666 | 0.278 | 0.359 | -0.293 | 0.331 |
| gamma2 | 0.091 | 0.768 | 0.297 | 0.324 | -0.394 | 0.183 |
| Total gamma | -0.110 | 0.721 | -0.292 | 0.332 | 0.356 | 0.233 |

PSG, polysomnography; EEG, electroencephalogram; Total delta, delta1 + delta2; Total beta, beta1 + beta2; Total gamma, gamma1 + gamma2. *^a^* Pearson’s correlation coefficient.

Table S3. Correlation between changes in PSG-EEG power ratio and changes in clinical symptoms in MDD patients *^a^*.

| Changes in PSG-EEG power ratio | Correlation coefficient | | | | | | | | | | | | | | |
| --- | --- | --- | --- | --- | --- | --- | --- | --- | --- | --- | --- | --- | --- | --- | --- |
|  | Changes in clinical symptoms (*n* = 13) | | | | | | | | | | | | | | |
|  | MADRS | *p* | HRSD-17 | *p* | HAMA | *p* | PSQI | *p* | | ISI | *p* | | CGI-S | *p* |  |
| delta1 | -0.197 | 0.518 | -0.255 | 0.400 | -0.403 | 0.172 | -0.234 | 0.441 | | 0.053 | 0.865 | | -0.341 | 0.254 |  |
| delta2 | 0.068 | 0.826 | 0.248 | 0.414 | 0.112 | 0.716 | 0.169 | 0.580 | | 0.416 | 0.157 | | 0.092 | 0.765 |  |
| Total delta | -0.142 | 0.645 | -0.198 | 0.517 | -0.348 | 0.244 | -0.198 | | 0.518 | 0.123 | | 0.688 | -0.271 | 0.371 |  |
| theta | -0.021 | 0.947 | 0.229 | 0.453 | 0.040 | 0.896 | 0.093 | 0.762 | | 0.345 | 0.249 | | 0.003 | 0.993 |  |
| alpha | 0.351 | 0.240 | 0.530 | 0.062 | 0.450 | 0.123 | 0.390 | 0.188 | | 0.245 | 0.420 | | 0.540 | 0.057 |  |
| beta1 | -0.211 | 0.488 | -0.283 | 0.349 | -0.076 | 0.805 | -0.185 | 0.546 | | -0.394 | 0.183 | | -0.223 | 0.463 |  |
| beta2 | -0.233 | 0.444 | -0.126 | 0.681 | -0.193 | 0.528 | -0.236 | 0.437 | | 0.100 | 0.745 | | 0.024 | 0.938 |  |
| Total beta | -0.185 | 0.544 | -0.247 | 0.417 | -0.027 | 0.931 | -0.154 | 0.615 | | -0.388 | 0.190 | | -0.169 | 0.580 |  |
| gamma1 | 0.495 | 0.085 | 0.485 | 0.093 | 0.606* | 0.028 | 0.463 | 0.111 | | 0.120 | 0.695 | | 0.691** | 0.009 |  |
| gamma2 | 0.502 | 0.081 | 0.442 | 0.131 | 0.557* | 0.048 | 0.437 | 0.135 | | 0.121 | 0.693 | | 0.659* | 0.014 |  |
| Total gamma | 0.505 | 0.078 | 0.466 | 0.109 | 0.585* | 0.036 | 0.453 | 0.120 | | 0.122 | 0.69 | | 0.681* | 0.010 |  |

PSG, polysomnography; EEG, electroencephalogram; HRSD-17, 17-item Hamilton Rating Scale for Depression; MADRS, Montgomery-Asberg Depression Rating Scale; HAMA, Hamilton Anxiety Scale; PSQI, Pittsburgh Sleep Quality Index; ISI, Insomnia Severity Index; CGI-S, Clinical Global Impressions-Severity.

Total delta, delta1 + delta2; Total beta, beta1 + beta2; Total gamma, gamma1 + gamma2. *^a^* Pearson’s correlation coefficient.

Table S4. Number of 30-s epochs used for EEG analysis

|  | Number of 30-s epochs used for EEG analysis |
| --- | --- |
| Health control | 1001.69 ± 90.32 |
| MDD patients (baseline) | 987.23 ± 75.64 |
| MDD patients (after 8 weeks of escitalopram treatment) | 975 ± 163.29 |
